# Impact of Obesity on the Clinical Profile of a Population-Based Sample with Chronic Obstructive Pulmonary Disease

**DOI:** 10.1371/journal.pone.0105220

**Published:** 2014-08-25

**Authors:** Francisco García-Rio, Joan B. Soriano, Marc Miravitlles, Luis Muñoz, Enric Duran-Tauleria, Guadalupe Sánchez, Victor Sobradillo, Julio Ancochea

**Affiliations:** 1 Servicio de Neumología, Hospital Universitario La Paz, IdiPAZ, Madrid, Spain, and CIBER de Enfermedades Respiratorias, Madrid, Spain; 2 Fundación Caubet-CIMERA Illes Balears, Bunyola, Illes Balears, Spain, and CIBER de Enfermedades Respiratorias, Illes Balears, Spain; 3 Pneumology Department, Hospital Universitario Vall d'Hebron, Barcelona, Spain; 4 Pneumology Department, Hospital Reina Sofía, Córdoba, Spain; 5 Centro de Investigación en Epidemiología Ambiental – CREAL, Barcelona, Spain; 6 Medical Department, GlaxoSmithkline S.A., Madrid, Spain; 7 Pneumology Department, Hospital de Cruces, Bilbao, Spain; 8 Servicio de Neumología, Hospital Universitario de La Princesa, Instituto de Investigación Sanitaria Princesa, Madrid, Spain; Clinica Universidad de Navarra, Spain

## Abstract

**Aims:**

To characterize the distribution of BMI in a population-based sample of COPD patients and to evaluate the impact of obesity on their health status, exercise tolerance, systemic inflammation and comorbidity.

**Methods:**

A population-based sample of 3,797 subjects aged 40–80 years from the EPI-SCAN study was selected. Subjects were categorized according their body mass index (BMI) as underweight (<18.5 kg/m^2^), normal weight (18.5–24.9 kg/m^2^), overweight (25.0–29.9 kg/m^2^) or obese (BMI≥30.0 kg/m^2^). Subjects were evaluated with post-bronchodilator spirometry and 6-minute walk tests. Smoking habits, respiratory symptoms, generic and specific quality of life, daily physical activities, comorbidities and systemic inflammatory biomarkers were recorded.

**Results:**

The prevalence of obesity or being overweight was higher in the 382 COPD patients than in the subjects without airflow limitation (29.4%, 95%CI 24.8–33.9% vs. 24.3, 95%CI 22.9–25.8; and 44.7%, 95%CI 39.7–49.6% vs. 43.0%, 95%CI 41.3–44.6, respectively; p = 0.020). In the COPD subgroup, obese subjects presented more dyspnea and less chronic cough, chronic bronchitis or chronic phlegm than normal-weight patients, as well as a worse health status. Moreover, reduced exercise tolerance and higher plasmatic C-reactive protein levels were found in the obese patients, who also presented a greater prevalence of cardiovascular disease (adjusted odds ratio 4.796, 95%CI 1.806–12.736, p = 0.002).

**Conclusions:**

In a population-based sample, obesity is more prevalent in COPD patients than in subjects without airflow limitation. Furthermore, obesity affects the clinical manifestations, quality of life and exercise tolerance of COPD patients, and it may contribute to a phenotype characterized by increased systemic inflammation and greater frequency of cardiovascular comorbidity.

## Introduction

Chronic obstructive pulmonary disease (COPD) is characterized by a range of pathophysiologic changes that contribute to a highly variable clinical presentation as well as heterogeneity among patients. In addition to airflow limitation, the integral evaluation of COPD needs to consider at least several aspects, such as symptoms, exacerbations and comorbidities [1], [2].

Comorbidities occur frequently in COPD patients but show great variability in reported prevalence [3]. COPD is often associated with significant nutritional abnormalities in particular. Extra-pulmonary manifestations such as low body weight, low body mass index (BMI) and depletion of fat-free mass have been extensively reported [4], [5], and they are recognized as independent prognostic factors in COPD patients [6], [7], often considered in the more advanced, late COPD stages and an indication of an emphysematous phenotype.

Although low BMI and muscle wasting have traditionally been the focus of nutritional assessment in COPD [7], recent data indicate that obesity is becoming frequent in this disease [8] and some evidence suggests that obesity might contribute to respiratory symptoms and exercise limitation, regardless of airflow obstruction [9]. However, the current evidence of a possible association between obesity and poorer COPD disease state is still limited.

Most studies concerning the prevalence of nutritional alterations in COPD have been performed in selected populations [10]–[12] and the impact of BMI on health status and prognosis seems to differ with COPD stage. In fact, in some studies, obese patients with more severe COPD appear to have better outcomes (“obesity paradox”) [6]. Little information is available regarding the BMI distribution in COPD patients from population-based studies [13], [14], which is necessary to more accurately represent the total spectrum of patients with the disease, and to provide more unbiased inferences.

We have analyzed the EPI-SCAN study [15], [16] aiming to characterize the BMI distribution in a population-based sample of COPD patients and to evaluate the impact of obesity on health status, exercise tolerance, daily physical activity, systemic inflammation and comorbidity.

## Materials and Methods

The study was approved by the corresponding ethics committees (Hospital Clinic, Barcelona, Spain). All participants gave written informed consent to participate in the study.

### Study population

We used data from the EPI-SCAN study, a multicenter, cross-sectional, population-based study conducted at 11 locations throughout Spain. Out of a total of 4,274 subjects who had been randomly contacted by telephone at the 11 sites, 3,885 agreed to participate in the study and a final group of 3,797 (88.8%) were available for analysis (complete minimum data set on gender, age and lung function). The 389 (9.1%) who refused to take part in the survey were slightly older and there were more women as well as never and former smokers. Complete details of the methodology, detailed descriptions of participation rates, and sample characteristics of the EPI-SCAN study have been published elsewhere [16]. For the present study, our source population included 3,797 non-institutionalized participants aged 40–80 who completed acceptable spirometry [17].

### Procedures

Body weight was assessed with a beam scale (measured to the nearest 0.1 kg) with subjects standing barefoot and in light clothing. Height was measured by a clinical stadiometer in bare or stocking feet. BMI, defined as weight (kg) divided by the square of height (meters), was calculated. Patients were categorized by World Health Organization (WHO) criteria as [18]: underweight (BMI<18.5 kg/m^2^), normal weight (BMI 18.5–24.9 kg/m^2^), overweight (BMI 25.0–29.9 kg/m^2^) and obese (BMI≥30.0 kg/m^2^).

Patient-reported data for smoking history, educational level, domestic and occupational exposures, respiratory history and symptoms, previous medication and use of health services during the preceding year were collected. Any respiratory exacerbation that required a change in regular medication was considered mild, while a respiratory exacerbation treated with a course of oral corticosteroids or antibiotics was considered moderate. Self-reported comorbidity was documented using the Charlson index. The presence of heart failure, ischemic heart disease, peripheral vascular disease or cerebrovascular disease was coded as cardiovascular disease.

Baseline dyspnea was assessed by the Modified Medical Research Council (mMRC) scale, and subjects completed the ECSC respiratory symptoms questionnaire, the Spanish versions of the London Chest Activity of Daily Living (LCADL) scale, the EQ-5D questionnaire and the St. George's Respiratory Questionnaire.

Blood samples were collected using standardized procedures and stored at −80°C for biomarker analysis, as previously described [19]. The biomarkers analyzed were: C-reactive protein (CRP), TNF-α, interleukin (IL)-6, IL-8, fibrinogen and nitrites/nitrates (NOx).

Pre- and post-bronchodilator spirometries were performed at each center using the same equipment and in accordance with current recommendations, as described in previous publications [15], [16]. A bronchodilator test was considered positive when there was an increase in FEV_1_ or FVC≥12% and ≥200 ml. Six-minute walk tests were performed following current guidelines [20]. To control for a learning effect, 2 walks were performed with a 30-minute rest in between. Walk-work was defined as 6-min walk distance × weight (in kg).

COPD was defined by a post-bronchodilator FEV_1_/FVC ratio <0.70 [1] and, in accordance with the new GOLD 2011 stage grades [1], COPD patients were classified in groups A (low risk, fewer symptoms), B (low risk, more symptoms), C (high risk, fewer symptoms) or D (high risk, more symptoms), using predicted FEV_1_, mMRC and exacerbation history. COPD severity was determined by the BODE [7] and ADO [21] indices. Subjects with a post-bronchodilator FEV_1_/FVC ratio ≥0.70 were considered not to have COPD.

### Statistical analysis

Values are expressed as mean ± standard deviation or count and percentage. Differences between study groups were analyzed using the Chi-squared test or ANOVA with post-hoc analysis by the Bonferroni test. In this analysis, a logarithmic transformation was used in those variables not normally distributed, to reduce their skewness. The effect of possible confounding factors was assessed using a generalized linear model analysis adjusted for age, gender, pack-years and post-bronchodilator FEV_1_ (% pred.).

In order to examine associations between variables, odds ratios were calculated by logistic regression. We developed multiple logistic regression models with adjustment for age, gender, pack-years and post-bronchodilator FEV_1_ (% pred.).

Analyses were performed with SPSS 14.0 for Windows (SPSS, Inc., Chicago, IL). A two-sided p value <0.05 was considered statistically significant.

## Results

**Figure 1:**
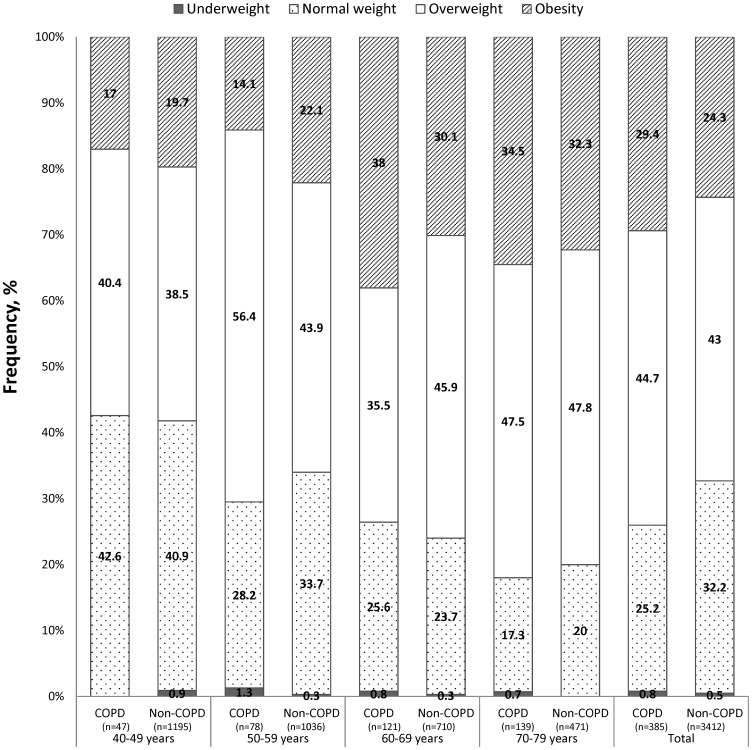
Distribution of BMI classifications according to age group in COPD and non-COPD subjects.

From the population-based sample of 3,797 subjects, 385 (10.1%) had post-bronchodilator FEV_1_/FVC<0.7. In comparison with the non-COPD subjects, COPD patients showed a higher prevalence of obesity (29.4%, 95%CI 24.8–33.9% vs. 24.3, 95%CI 22.9–25.8) and of being overweight (44.7%, 95%CI 39.7–49.6% vs. 43.0%, 95% CI 41.3–44.6%) (p = 0.020). In the global sample, being underweight was very uncommon, both in COPD patients (0.8%, 95% CI 0.0–1.7%) as well as in non-COPD subjects (0.5%, 95%CI 0.2–0.7%). Figure 1 shows the BMI distribution in the COPD and non-COPD subjects according to age strata.

Table 1 demonstrates the main characteristics of the normal-weight, overweight and obese COPD groups. In comparison with the normal-weight COPD patients, overweight and obese patients were older and had greater smoking intensity, whereas no differences in gender, occupational exposure or education level were found. FVC and FEV_1_ were slightly lower in obese COPD patients than in the other groups, without significant differences in inspiratory capacity or reversibility. Moreover, obese COPD patients walked less distance during six minutes, with a higher walk work than COPD patients with normal weight or overweight. Similarly, comorbidities were more frequent in obese COPD patients than in the other two groups, often associated with the presence of cardiovascular disease or diabetes mellitus. GOLD 2011 grade distribution was also different among the COPD patient groups. Obese COPD patients also showed higher multidimensional severity indices than the other groups and they more frequently used respiratory medication, specifically beta-agonists, methylxanthines and inhaled corticosteroids.

**Table 1:** General characteristics, functional status and severity of the COPD groups.

	Normal weight	Overweight	Obesity	Total	p
	(18.5–24.9 Kg/m^2^)	(25–29.9 Kg/m^2^)	(>30 Kg/m^2^)		
Patients, n	97	172	113	382	
Males, %	63.9%	75.6%	68.1%	70.4%	0.108
Age, yr.	61±10	64±11^‡^	66±9^†^	64±10	0.001
BMI, Kg/m^2^	22.9±1.7	27.4±1.5	33.7±3.7	28.1±4.7	<0.001
Smoking status					<0.001
Current smoker, %	50.5	33.7	19.5	33.8	
Former smoker, %	33.0	39.0	48.7	40.3	
Never smoker, %	16.5	27.3	31.9	25.9	
Smoking exposure, pack-years	34.1±21.6	43.3±29.1^‡^	46.1±24.9^‡^	41.4±26.3	0.009
Self-reported exposure to vapors, gases, dusts or fumes, %	38.1	34.9	43.4	38.2	0.354
Occupational exposure					
Biological dusts, %	25.8	19.8	30.1	24.3	0.130
Mineral dusts, %	28.9	24.4	25.7	25.9	0.725
Gases or fumes, %	25.8	15.7	24.8	20.9	0.073
Retired or disabled, %	67.0	72.7	87.5	75.6	0.001
Education level					0.096
Less than primary school, %	14.6	14.0	20.4	16.0	
Primary school, %	34.4	37.2	47.8	39.6	
Secondary school, %	25.0	27.3	21.2	24.9	
University degree, %	24.0	19.8	10.6	18.1	
Pulmonary function					
Post-bronchodilator FVC, % pred.	110±20	107±21	100±21^†§^	106±21	0.002
Post-bronchodilator FEV_1_, % pred.	85±20	85±19	79±19	83±20	0.037
Post-bronchodilator FEV_1_/FVC, %	62±9	62±7	62±7	62±8	0.976
Post-bronchodilator IC, % pred.	103±28	109±26	106±27	106±27	0.239
Reversibility (positive bronchodilator test) (%)	20.2	31.0	25.9	26.8	0.161
6-min walk test					
Distance, m	468±122	460±114	405±131^†¶^	447±123	0.001
Walk work (m. Kg)	30723±8645	36152±10280^†^	39329±12504^†¶^	35251±10912	<0.001
Δ SpO_2_, %	−1.13±2.09	−0.69±2.02	−1.01±3.35	−0.90±2.49	0.377
Δ Borg	0.87±1.22	0.78±1.12	0.98±1.24	0.86±1.17	0.451
Δ Borg/distance walked, %/100 m	0.23±43	0.19±0.32	0.36±0.93	0.25±0.59	0.085
Comorbidity					
Cardiovascular diseases, %	9.3	15.7	31.9	18.8	<0.001
Diabetes mellitus, %	7.2	9.3	19.5	11.8	0.009
Peptic ulcer disease, %	10.3	7.6	5.3	7.6	0.395
Neoplasm, %	7.2	6.4	5.3	6.3	0.848
Charlson index	0.90±1.01	0.84±0.96	1.29±1.15^‡¶^	0.99±1.04	0.001
GOLD risk stage					<0.001
A, %	66.0	59.3	33.6	53.4	
B, %	20.6	32.0	47.8	33.8	
C, %	2.1	1.2	4.4	2.4	
D, %	11.3	7.6	14.2	10.5	
BODE index	1.10±1.55	0.86±1.23	1.61±1.78^§^	1.13±1.53	0.001
ADO index	2.28±1.69	2.58±1.64	3.31±1.52^†¶^	2.72±1.66	<0.001
COPD previous diagnosis, %	29.9	20.9	33.6	27.0	0.046
Current treatment					
Short-acting beta-agonists, %	13.4	11.6	27.4	16.9	0.001
Long-acting beta-agonists, %	17.5	17.4	30.1	21.2	0.023
Anticholinergics, %	14.4	11.0	20.4	14.7	0.094
Methylxanthines, %	2.1	0	4.4	1.8	0.024
Inhaled corticosteroids, %	18.6	19.8	31.9	23.0	0.029

Values are mean ±SD or frequency. Abbreviations: BMI = body mass index; FVC = forced vital capacity; FEV_1_ = forced expiratory volume in 1 second; IC = inspiratory capacity; SpO_2_ = oxyhemoglobin saturation.

Comparisons between groups by ANOVA with Bonferroni post-hoc comparisons: ^†^p<0.01 vs. normal weight group; ^‡^p<0.05 vs. normal weight group; ^¶^p<0.01 vs. overweight group; ^§^p<0.05 vs. overweight group.

The comparison of symptoms, exacerbation history, health status and systemic inflammatory biomarkers of the three study groups are presented in table 2. Dyspnea and wheezing were more frequent in obese COPD patients, while chronic bronchitis and phlegm resulted more frequent in COPD patients with normal weight. Significant differences in health-related quality of life, daily physical activity and inflammatory biomarker levels were noted among the three groups. In the adjusted comparison, both dyspnea and health status were worse in obese COPD patients than in overweight COPD patients (Table 3). Reduced exercise tolerance and higher walk work were found in obese patients, although daily physical activity did not reach significant differences between the study groups. Finally, systemic pro-inflammatory state was increased in obese or overweight COPD patients compared with COPD patients with normal weight, showing higher plasmatic CRP levels. Mild and moderate exacerbations and hospitalizations were not significantly different among the three groups.

**Table 2:** Comparison of symptoms, previous exacerbations, health status and systemic biomarker levels between the study groups.

	Normal weight	Overweight	Obesity	Total	p
	(18.5–24.9 Kg/m^2^)	(25–29.9 Kg/m^2^)	(>30 Kg/m^2^)		
Symptoms					
Chronic cough, %	39.6	32.0	25.0	31.8	0.079
Chronic bronchitis, %	29.9	18.6	12.6	19.7	0.007
Chronic phlegm, %	39.2	31.4	21.4	30.4	0.020
Dyspnea, %	20.6	23.4	44.2	28.9	<0.001
mMRC	1.49±0.86	1.52±0.75	1.92±0.91^†¶^	1.63±0.84	<0.001
Wheezing, %	64.9	58.1	72.6	64.1	0.045
Mild respiratory exacerbations in the previous year, n per patient	0.10±0.47	0.30±1.77	0.31±0.80	0.25±1.29	0.411
Moderate respiratory exacerbations in the previous year, n per patient	0.45±1.07	0.33±1.05	0.48±0.81	0.40±0.99	0.380
Hospitalizations in the previous year, n per patient	0.02±0.20	0.06±0.41	0.06±0.41	0.05±0.37	0.613
Health-related quality of life					
SGRQ symptoms	29.1±22.8	23.8±21.1	29.2±22.5	26.8±22.1	0.088
SGRQ activity	26.1±27.0	25.6±23.1	38.7±24.9^†¶^	29.5±25.3	<0.001
SGRQ impact	14.6±18.7	12.0±15.1	16.6±17.2	14.0±16.8	0.109
Total SGRQ	20.7±20.2	18.4±16.5	25.7±18.1^¶^	21.1±18.2	0.008
EQ-5D VAS score	71.6±16.7	73.1±15.6	66.7±18.6^§^	70.8±17.0	0.013
EQ-5D utility score	0.83±0.24	0.90±0.19	0.83±0.21	0.86±0.21	0.022
LCADL total	17.1±8.1	15.6±4.1	17.7±7.2^§^	16.6±6.4	0.030
Systemic biomarkers					
CRP, log (mg/l)	0.37±0.31	0.51±0.45	0.60±0.40^†^	0.50±0.41	0.003
TNF-alpha, log (pg/ml)	0.97±0.27	1.06±0.28^‡^	1.06±0.52	1.04±0.27	0.036
IL-6, log (pg/ml)	0.48±0.34	0.46±0.34	0.54±0.35	0.49±0.34	0.216
IL-8, log (pg/ml)	0.51±0.64	0.43±0.60	0.41±0.55	0.44±0.60	0.482
Fibrinogen, g/l	3.24±0.91	3.57±1.13	3.77±1.08^†^	3.54±1.08	0.005
NOx, log (nmol/l)	1.43±0.18	1.42±0.25	1.40±0.22	1.42±0.23	0.625

Values are mean ± SD or frequency. Abbreviations: BMI = body mass index; mMRC = modified Medical Research Council dyspnea scale; SGRQ = St. George's Respiratory Questionnaire; EQ-5D = EuroQol 5 Dimensions questionnaire; VAS = Visual analogue scale; LCADL = London Chest Activities of Daily Living; CRP = C-reactive protein; TNF = tumor necrosis factor; IL = interleukin; NOx = nitrites/nitrates.

Comparisons between groups by ANOVA with Bonferroni post-hoc comparisons: ^†^p<0.01 vs. normal weight group; ^‡^p<0.05 vs. normal weight group; ^¶^p<0.01 vs. overweight group; ^§^p<0.05 vs. overweight group.

**Table 3:** Adjusted comparisons of health status, exercise tolerance and systemic inflammatory biomarkers between the study groups.

	Normal weight	Overweight	Obesity	p
	(18.5–24.9 Kg/m^2^)	(25–29.9 Kg/m^2^)	(>30 Kg/m^2^)	
mMRC	1.58±0.09	1.48±0.07	1.81±0.09^§^	0.017
Health-related quality of life				
SGRQ symptoms	29.3±2.6	24.5±2.0	26.5±2.6	0.332
SGRQ activity	28.4±2.8	24.9±2.2	35.9±2.8^¶^	0.008
SGRQ impact	15.2±1.9	11.6±1.5	14.6±2.0	0.250
Total SGRQ	21.8±2.1	18.0±1.6	23.3±2.1	0.098
EQ-5D VAS score	71.3±1.9	74.0±1.5	67.9±2.0^§^	0.048
EQ-5D utility score	0.82±0.02	0.91±0.02	0.86±0.03^‡^	0.017
LCADL total	17.3±0.7	15.4±0.6	16.8±0.7	0.097
Distance 6-min walking test, m	450±14	471±11	422±15^§^	0.043
Walk work, m. Kg	28144±1246	36815±981^†^	40232±1302^†^	<0.001
Systemic biomarkers				
CRP, log (mg/l)	0.40±0.05	0.54±0.04^‡^	0.57±0.05^‡^	0.033
TNF-alpha, log (pg/ml)	0.97±0.03	1.05±0.03	1.03±0.04	0.218
IL-6, log (pg/ml)	0.48±0.04	0.48±0.03	0.57±0.04	0.231
IL-8, log (pg/ml)	0.53±0.07	0.42±0.06	0.42±0.07	0.416
Fibrinogen, g/l	3.4±0.1	3.5±0.1	3.6±0.1	0.431
Albumin, g/l	45.2±0.3	45.4±0.3	46.0±0.4	0.188
NOx, log (nmol/l)	1.44±0.03	1.44±0.02	1.41±0.03	0.589

Values are mean ± SEM. Comparisons adjusted by gender, age, pack-year and FEV_1_ (% pred). Abbreviations: mMRC = modified Medical Research Council dyspnea scale; SGRQ = St. George's Respiratory Questionnaire; EQ-5D = EuroQol 5 Dimensions questionnaire; VAS = Visual analogue scale; LCADL = London Chest Activities of Daily Living; CRP = C-reactive protein; TNF = tumor necrosis factor; IL = interleukin; NOx = nitrites/nitrates.

Post-hoc comparisons between groups by Bonferroni test: ^†^p<0.001 vs. normal weight group; ^‡^p<0.05 vs. normal weight group; ^¶^p<0.01 vs. overweight group; ^§^p<0.05 vs. overweight group.

**Figure 2:**
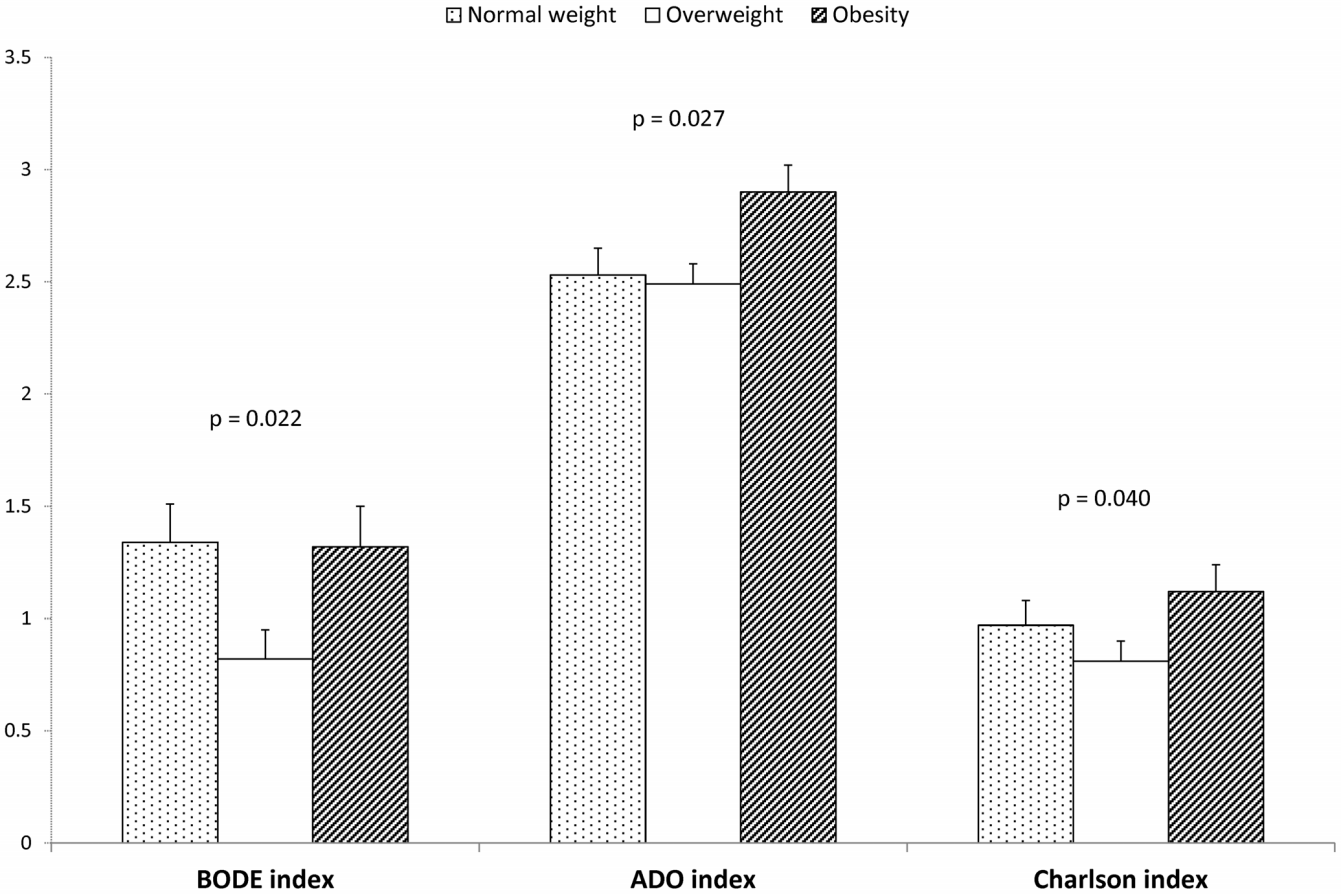
Adjusted comparisons of multidimensional indices and comorbidity between the three groups of COPD patients. Mean values are adjusted for gender, age, packs-year and FEV_1_ (% pred.). Error bars represent SEM.

After multivariate adjustment, differences in multidimensional COPD assessment and in the Charlson index remained among the three study groups (Figure 2). Consistently, BODE and ADO indices were higher in obese COPD patients than in overweight COPD subjects. In turn, comorbidities were more frequent in the obese patients than in the other two COPD groups.

**Figure 3:**
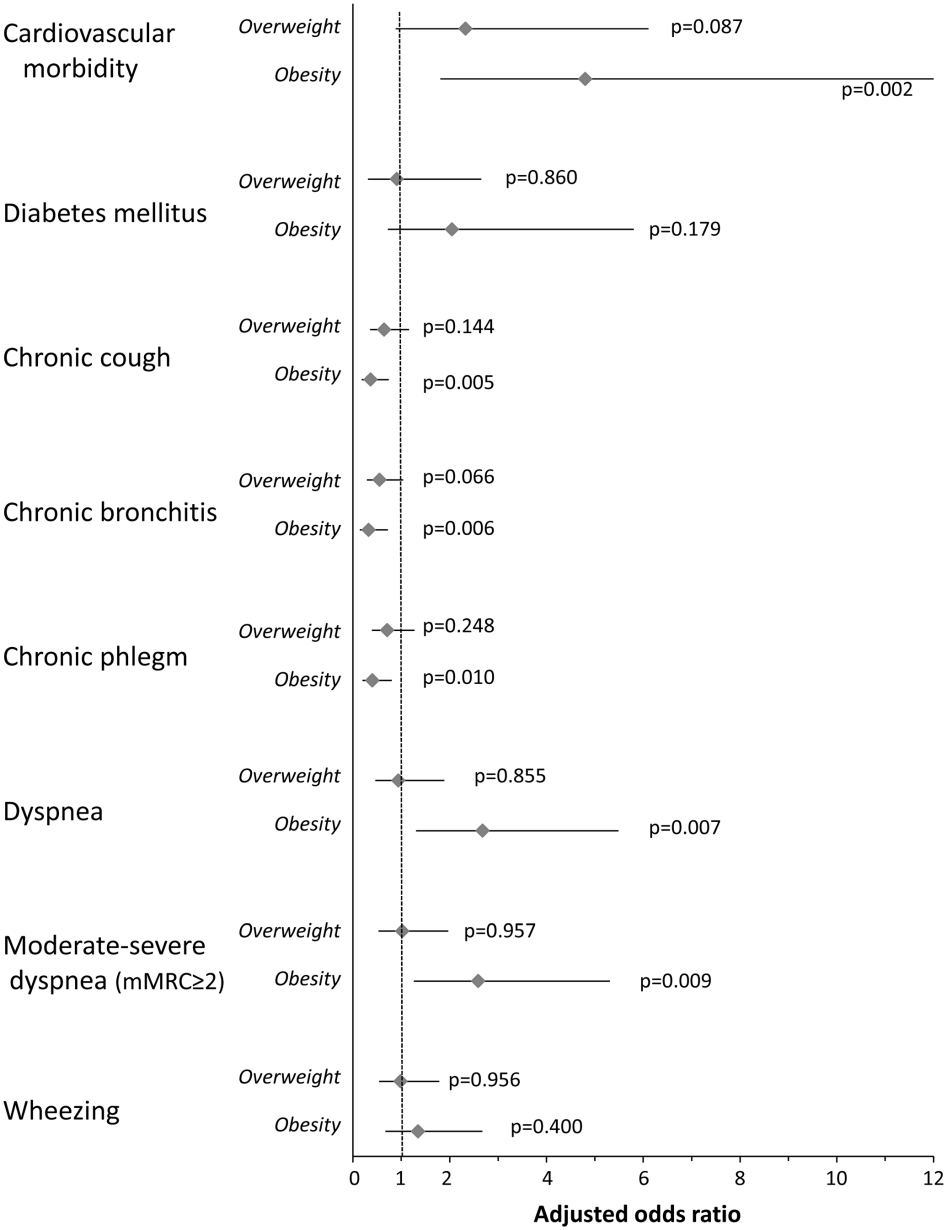
Adjusted risk of comorbidity and respiratory symptoms in overweight and obese COPD patients compared with normal-weight COPD patients. Values are adjusted for gender, age, packs-year and FEV_1_ (% pred.).

Adjusted odds ratios for comorbidity and respiratory symptoms by COPD group are shown in Figure 3. In comparison with normal-weight COPD patients, the obese patients presented a greater prevalence of cardiovascular diseases (adjusted odds ratio [OR] 4.796, 95%CI 1.806–12.736, p = 0.002) and a different symptomatic profile. The obese COPD patients had lower risk for presenting chronic cough (adjusted OR 0.364, 95% CI 0.178–0.743, p = 0.005), chronic bronchitis (adjusted OR 0.321, 95%CI 0.142–0.724, p = 0.006) or chronic phlegm (adjusted OR 0.398, 95%CI 0.197–0.804, p = 0.010) than COPD patients with normal weight, whereas they had more dyspnea (adjusted OR 2.679, 95%CI 1.307–5.489, p = 0.007).

## Discussion

Our study shows that being underweight is uncommon in a population-based series of COPD patients, while obesity affects more than 29% of patients and is more frequent than in subjects without airflow limitation. Also, the presence of obesity determines the clinical behavior of COPD, with more prevalence and severity of dyspnea and less frequency of cough and expectoration, worse quality of life, reduced exercise tolerance, more systemic inflammation and major cardiovascular comorbidity.

Although a previous population study [13] reported a slightly higher incidence (6.7%) of underweight patients, it used a different cut-point than that established by the WHO (BMI<20 Kg/m^2^). Our results concur with a series from the US Department of Veterans Affairs Puget Sound Health Care System, which reported only 1.1% of patients with a BMI<18.5 Kg/m^2^ [22]. In another study, a prevalence of self-reported underweight lower than 4% has been reported [14]. With regards to obesity, the prevalence observed in our COPD patients (29%) concurs with a large Canadian pulmonary function laboratory database (29%) [23], the PLATINO study (23%) [13] and Canadian National Health Survey (24.6%) studies [14]. Moreover, our data confirm that obesity is more prevalent in patients with COPD than in the general population, which probably depends on the severity of chronic airflow limitation. In fact, there is evidence for higher prevalence of obesity in patients with early stage COPD [9] and it has been reported that obesity is more prevalent in COPD patients with mild obstruction than in severe patients [24]. It is likely that the correlation with severity justifies both the high prevalence of obesity as well as the very low incidence of underweight patients in population studies in which the patient group is mainly comprised of subjects with mild-moderate disease.

The three COPD groups (normal weight, overweight and obese) compared in our study were not homogenous in age or smoking history. Obese patients were older, probably reflecting the physiological weight increase that happens with age and sedentarism associated with exercise- and effort-induced dyspnea [25]. Moreover, and in agreement with Cecere et al. [22], obese patient groups had a lower frequency of current smokers, although the cumulative smoking exposure was higher, probably due to an age effect.

As for lung function, the FVC and FEV_1_ of our obese patients were lower than those of normal-weight patients. These findings, which have also been identified in other studies [22], [26], coincide with common physiological abnormalities classically associated with simple obesity, such as decreased chest wall and lung compliance, small airway dysfunction and expiratory flow limitation [27]. Contrarily, the lack of differences in inspiratory capacity among our patient groups contradicts with previous information from O'Donnell et al. [23], who reported a directly proportional relationship between BMI and resting IC, suggesting that obesity might reduce lung hyperinflation. This discordance could be attributed to the differing severity and age of the patients included in the two studies, and particularly to the fact that in the O'Donnell study the obese patients were younger than the patients with normal weight, while in our study the opposite was true.

Possibly one of the most prominent findings of our study lies in the impact of obesity on the symptoms profile of COPD patients, where patients with obesity had more dyspnea and less cough and expectoration than patients with normal weight. An analysis of the PLATINO study [13] also reported a higher frequency of dyspnea and wheezing in obese patients, although there were no variations in cough or phlegm. In addition to being more frequent, dyspnea is also more intense in obese patients compared with normal-weight patients, even after adjusting for airflow limitation and other variables. Compared with a small clinical series of COPD patients [28] that identified lower baseline dyspnea in obese patients, which was attributed to lower resting hyperinflation, other more extensive clinical series confirm that obese patients with COPD more frequently experience moderate or severe dyspnea than normal-weight patients [22], [26]. A greater perception of the dyspnea associated with obesity may contribute to this finding [29]. Moreover, obesity might differ between various clinical phenotypes of COPD, as suggested in the Tucson prospective cohort study [8].

The crude comparison between the patient groups shows that obese COPD patients presented a greater use of respiratory medication and a poorer health-related quality of life. In the adjusted analysis, however, only the EuroQoL showed significant differences between groups, while the SGRQ did not reach statistical significance, possibly reflecting the contribution of other determinants (that are not exclusively respiratory in nature) to the poorer quality of life of obese patients.

An aspect that is particularly important is the potential effect of obesity on the perception of symptoms of patients with asthma-COPD overlap syndrome. Taking into account the fact that obesity notably contributes to the breathlessness of asthma patients [30], [31] and that some 17% of patients with COPD in our study were categorized as asthma-COPD phenotype [32], one could speculate that the clinical impact of obesity was provided exclusively by this patient subgroup. To assess this possibility, Tables S1-S4 in File S1 present the comparisons between groups while excluding patients with asthma-COPD overlap syndrome, which demonstrates that the effects of obesity on clinical manifestations, quality of life, exercise tolerance and systemic inflammation persist in the remaining COPD patients.

There is a certain discrepancy around the effect of obesity on exercise tolerance in COPD. While reduced tolerance to exercise has been reported [23], other authors do not identify such effect [28], [33]. Our results confirm that adjusted exercise tolerance is lower in obese patients who reach a shorter distance walked during the 6-minute walk test. Although the mechanisms of exercise intolerance in obese patients with COPD may be complex due to disease heterogeneity and the existence of serious comorbidities, the neuromechanical uncoupling of the respiratory system seems to play some type of role. Compared to normal-weight COPD patients, obese patients have a higher metabolic demand at any given power output as a result of the high oxygen cost of lifting heavy limbs [23]. Moreover, obese patients with COPD have increased ventilatory demand and more frequently abnormal ventilatory mechanics. In fact, ventilatory requirements are higher in obese COPD patients compared with those of normal weight as a result of combined increased metabolic loading, high fixed physiological dead space and possibly earlier metabolic acidosis [28]. Given the increase in ventilatory demand and the mechanical limitation of ventilation, the muscular effort required to sustain ventilation during exercise is also higher [23]. The net effect of these abnormalities is the increased work and oxygen cost of breathing at any given power output compared with normal-weight individuals, which would be expected to contribute to exercise limitation. This hypothesis coincides with our results that obese patients develop greater walk-work, defined as 6 min walk distance × weight (in kg), and the results of others who demonstrated that obese COPD patients had higher peak symptom-limited oxygen uptake than non-obese patients [28], [34].

In the general population, obesity is a strong risk factor for the development and progression of hypertension, dyslipidemia, metabolic syndrome, and type-2 diabetes mellitus [35]. Our results confirm this effect in COPD patients and that the presence of obesity almost multiplies five-fold the risk for presenting cardiovascular comorbidity. In this sense, it has been reported that the application of cluster analysis to segregate COPD patients into different phenotypes has been able to identify a group characterized by obesity and cardiovascular disease [36]. This association is particularly relevant because several studies have shown that cardiovascular diseases, along with lung cancer, are among the main causes of death in COPD patients not selected by severity criteria [37], [38]. In spite of this, there continues to be controversy about the potential protective effect of obesity in COPD. Although it has been shown that, in patients with COPD, being overweight or obese had a protective effect against mortality [39], an analysis of the NHANES III cohort has reported that obesity is associated with increased mortality among participants with obstructive lung disease [40]. Furthermore, the increase in dyspnea induced by obesity could worsen the prognosis of these patients. In fact, it has been reported that persistent dyspnea and dyspnea development are risk factors for all-cause, cardiovascular and respiratory mortality in COPD patients, and that dyspnea effects on mortality are more pronounced in obese and older subjects [41].

Our results give supporting data to link obesity with the systemic inflammatory COPD phenotype. In COPD patients, the association between circulating CRP levels and BMI has previously been demonstrated [42]. Is has also been shown that the presence of elevated plasma CRP concentrations is associated with increased risk for the development of cardiovascular comorbidity [43]. From the PAC-COPD study, one of the three COPD subtypes included patients with milder airflow limitation and a high proportion of obesity, cardiovascular disorders, diabetes and systemic inflammation [36]. Most of the features of the so-called metabolic syndrome, including obesity, higher levels of triglycerides, diabetes, ischemic heart disease, arterial hypertension and elevated serum levels of C-reactive protein and fibrinogen, were clustered in this COPD subtype. So, obesity might be a marker for the proposed “chronic systemic inflammatory syndrome”, which contributes to the comorbidities that frequently co-exist in patients with COPD [44].

Our study, however, presents several limitations. The evaluation of the nutritional state of the patients was based on BMI and we did not have any information regarding body composition. We do not have a longitudinal follow-up of the patients; therefore, it was not possible to evaluate the relationship between obesity throughout the progression of the disease. Furthermore, our sample presents a very small number of patients with severe or very severe disease because it provides a representative sample of the population distribution seen in COPD, with a predominance of milder patients.

To conclude, our study provides evidence of the higher prevalence of obesity in a population-based sample of patients with COPD. Moreover, our results show that obesity affects the clinical manifestations, quality of life and exercise tolerance of COPD patients, and that obesity might help define a phenotype characterized by increased systemic inflammation and greater frequency of cardiovascular comorbidity.

## Supporting Information

File S1 **Comparisons between COPD subgroups excluding patients with asthma-COPD overlap syndrome.** Combined file includes Table S1-S4. **Table S1**. General characteristics, functional status and severity of the COPD groups excluding the patients with mixed COPD-asthma phenotype. **Table S2**. Comparison of symptoms, previous exacerbations, health status and systemic biomarker levels between the study groups excluding the patients with mixed COPD-asthma phenotype. **Table S3**. Adjusted comparisons of health status, exercise tolerance and systemic inflammatory biomarkers between the study groups excluding the patients with mixed COPD-asthma phenotype. **Table S4**. Adjusted risk of comorbidity and respiratory symptoms in overweight and obese COPD patients compared with normal-weight COPD patients excluding the subjects with mixed phenotype COPD-asthma. Values are adjusted for gender, age, packs-year and FEV1 (% pred.).
(DOCX)
