# Supplementary material for: Impact of Obesity on the Clinical Profile of a Population-Based Sample with Chronic Obstructive Pulmonary Disease
Source: PLoS One. 2014 Aug 25;9(8):e105220. doi: 10.1371/journal.pone.0105220 (PMC4143213; doi:10.1371/journal.pone.0105220)
Supplement: File S1 — Comparisons between COPD subgroups excluding patients with asthma-COPD overlap syndrome. Combined file includes Table S1-S4. Table S1. General characteristics, functional status and severity of the COPD groups excluding the patients with mixed COPD-asthma phenotype. Table S2. Comparison of symptoms, previous exacerbations, health status and systemic biomarker levels between the study groups excluding the patients with mixed COPD-asthma phenotype. Table S3. Adjusted comparisons of health status, exercise tolerance and systemic inflammatory biomarkers between the study groups excluding the patients with mixed COPD-asthma phenotype. Table S4. Adjusted risk of comorbidity and respiratory symptoms in overweight and obese COPD patients compared with normal-weight COPD patients excluding the subjects with mixed phenotype COPD-asthma. Values are adjusted for gender, age, packs-year and FEV1 (% pred.). (DOCX) [file pone.0105220.s001.docx]

Title: **Impact of obesity on the clinical profile of a population-based sample with chronic obstructive pulmonary disease**

Authors: Francisco García-Rio^1^, MD; Joan B Soriano^2^, MD; Marc Miravitlles^3^, MD; Luis Muñoz^4^, MD; Enric Duran-Tauleria^5^, MD; Guadalupe Sánchez^6^, MD; Victor Sobradillo^7^, MD; Julio Ancochea^8^, MD

**S1. Supporting Information**

**Table S1**. General characteristics, functional status and severity of the COPD groups excluding the patients with mixed COPD-asthma phenotype

|  | | **Normal weight**  (18.5-24.9 Kg/m^2^) | **Overweight**  (25-29.9 Kg/m^2^) | **Obesity**  (>30 Kg/m^2^) | **Total** | **p** |
| --- | --- | --- | --- | --- | --- | --- |
| Patients, n | | 86 | 144 | 85 | 315 |  |
| Males, % | | 68.6 | 79.9 | 75.3 | 75.6 | 0.157 |
| Age, yr. | | 61 ± -10 | 64 ± 10 ‡ | 67 ± 9 † | 64 ± 10 | 0.001 |
| BMI, Kg/m^2^ | | 22.8 ± 1.7 | 27.4 ± 1.5 † | 33.8 ± 4.0 †¶ | 27.9 ± 4.7 | <0.001 |
| Smoking status | | | | | | <0.001 |
|  | Current smoker, % | 55.8 | 35.4 | 22.4 | 37.5 |  |
|  | Former smoker, % | 32.6 | 42.4 | 54.1 | 42.9 |  |
|  | Never smoker, % | 11.6 | 22.2 | 23.5 | 19.7 |  |
| Smoking exposure, pack-years | | 35 ± 21 | 45 ± 30 ‡ | 48 ± 25 ‡ | 43 ± 27 | 0.007 |
| Self-reported exposure to vapors, gases, dusts or fumes, % | | 39.5 | 30.6 | 48.2 | 37.8 | 0.026 |
| Occupational exposure | |  |  |  |  |  |
|  | Biological dusts, % | 25.6 | 18.1 | 32.9 | 24.1 | 0.037 |
|  | Mineral dusts, % | 29.1 | 22.2 | 25.9 | 25.1 | 0.501 |
|  | Gases or fumes, % | 25.6 | 14.6 | 27.1 | 21.0 | 0.038 |
| Retired or disabled, % | | 65.1 | 72.9 | 88.2 | 74.9 | 0.002 |
| Education level | | | | | | 0.118 |
|  | Less than primary school, % | 15.3 | 11.1 | 21.2 | 15.0 |  |
|  | Primary school, % | 32.9 | 34.7 | 44.7 | 36.9 |  |
|  | Secondary school, % | 24.7 | 29.2 | 22.4 | 26.1 |  |
|  | University degree, % | 24.7 | 22.9 | 11.8 | 20.4 |  |
| Pulmonary function | | | | | | |
|  | Post-bronchodilator FVC, % pred. | 110 ± 20 | 107 ± 20 | 100 ± 22 †§ | 106 ± 21 | 0.005 |
|  | Post-bronchodilator FEV_1_, % pred. | 86 ± 20 | 85 ± 19 | 79 ± 20 | 84 ± 20 | 0.060 |
|  | Post-bronchodilator FEV_1_/FVC, % | 62 ± 9 | 62 ± 7 | 62 ± 7 | 62 ± 8 | 0.994 |
|  | Post-bronchodilator IC, % pred. | 103 ± 28 | 109 ± 26 | 106 ± 27 | 106 ± 27 | 0.239 |
|  | Reversibility (positive bronchodilator test) (%) | 19.0 | 32.9 | 22.2 | 26.3 | 0.046 |
| 6-min walk test | | | | | | |
|  | Distance, m | 464 ± 119 | 462 ± 117 | 400 ± 115 †¶ | 447 ± 120 | 0.001 |
|  | Walk work (m . Kg) | 294 ± 87 | 357 ± 104 † | 360 ± 120 † | 340 ± 108 | <0.001 |
|  | Δ SpO_2_, % | -1.0 ± -2.0 | -0.8 ± 2.0 | -0.8 ± 3.4 | -0.9 ± 2.5 | 0.818 |
|  | Δ Borg | 0.8 ± 1.2 | 0.7 ± 1.0 | 1.0 ± 1.3 | 0.8 ± 1.2 | 0.334 |
|  | Δ Borg/distance walked, %/100 m | 22 ± 45 | 17 ± 27 | 37 ± 102 | 24 ± 61 | 0.087 |
| Comorbidity | | | | | | |
|  | Cardiovascular diseases, % | 7.0 | 16.7 | 32.9 | 18.4 | <0.001 |
|  | Diabetes mellitus, % | 8.1 | 7.6 | 18.8 | 10.8 | 0.020 |
|  | Peptic ulcer disease, % | 11.6 | 6.9 | 7.1 | 8.3 | 0.411 |
|  | Neoplasm, % | 7.0 | 6.9 | 7.1 | 7.0 | 0.999 |
| Charlson index | | 0.8 ± 1.0 | 0.7 ± 0.9 | 1.2 ± 1.2 ‡¶ | 0.9 ± 1.1 | 0.002 |
| GOLD risk stage | | | | | | <0.001 |
|  | A, % | 70.9 | 63.2 | 35.3 | 57.8 |  |
|  | B, % | 17.4 | 30.6 | 48.2 | 31.7 |  |
|  | C, % | 2.3 | 0.7 | 3.5 | 1.9 |  |
|  | D, % | 9.3 | 5.6 | 12.9 | 8.6 |  |
| BODE index | | 1.07 ± 1.56 | 0.82 ± 1.21 | 1.55 ± 1.63 ¶ | 1.08 ± 1.45 | 0.004 |
| ADO index | | 2.28 ± 1.75 | 2.53 ± 1.56 | 3.29 ± 1.47 †¶ | 2.67 ± 1.63 | <0.001 |
| COPD previous diagnosis, % | | 32.6 | 21.5 | 37.96 | 28.9 | 0.023 |
| Current treatment | | | | | | |
|  | Short-acting beta-agonists, % | 9.3 | 7.6 | 15.3 | 10.2 | 0.171 |
|  | Long-acting beta-agonists, % | 11.6 | 10.4 | 20.0 | 13.3 | 0.103 |
|  | Anticholinergics, % | 14.0 | 10.4 | 18.8 | 13.7 | 0.200 |
|  | Methylxanthines, % | 2.3 | 0 | 2.4 | 1.3 | 0.182 |
|  | Inhaled corticosteroids, % | 12.8 | 13.2 | 20.0 | 14.9 | 0.305 |

Values are mean ± SD or frequency. Abbreviations: BMI=body mass index; FVC=forced vital capacity; FEV_1_=forced expiratory volume in 1 second; IC=inspiratory capacity; SpO_2_=oxyhemoglobin saturation.

Comparisons between groups by ANOVA with Bonferroni post-hoc comparisons: † p<0.01 vs. normal weight group; ‡ p<0.05 vs. normal weight group; ¶ p<0.01 vs. overweight group; § p<0.05 vs. overweight group.

**Table S2**. Comparison of symptoms, previous exacerbations, health status and systemic biomarker levels between the study groups excluding the patients with mixed COPD-asthma phenotype

|  | | **Normal weight**  (18.5-24.9 Kg/m^2^) | **Overweight**  (25-29.9 Kg/m^2^) | **Obesity**  (>30 Kg/m^2^) | **Total** | **p** |
| --- | --- | --- | --- | --- | --- | --- |
| Symptoms | | | | | | |
|  | Chronic cough, % | 38.4 | 25.0 | 20.2 | 27.4 | 0.020 |
|  | Chronic bronchitis, % | 22.1 | 11.8 | 20.0 | 16.8 | 0.086 |
|  | Chronic phlegm, % | 30.6 | 19.4 | 16.5 | 21.7 | 0.036 |
|  | Dyspnea, % | 26.7 | 36.1 | 61.2 | 40.3 | <0.001 |
|  | mMRC | 1.47 ± 0.89 | 1.46 ± 0.72 | 1.89 ± 0.89 †¶ | 1.58 ± 0.84 | <0.001 |
|  | Wheezing, % | 61.6 | 52.1 | 65.9 | 58.4 | 0.096 |
| Mild respiratory exacerbations in the previous year, n per patient | | 0.06 ± 0.32 | 0.10 ± 0.36 | 0.27 ± 0.73 ‡§ | 0.13 ± 0.49 | 0.008 |
| Moderate respiratory exacerbations in the previous year, n per patient | | 0.40 ± 1.07 | 0.19 ± 0.57 | 0.39 ± 0.69 § | 0.30 ± 0.77 | 0.076 |
| Hospitalizations in the previous year, n per patient | | 0.02 ± 0.22 | 0.03 ± -0.28 | 0.07 ± -0.46 | 0.04 ± 0.32 | 0.597 |
| Health-related quality of life | | | | | | |
|  | SGRQ symptoms | 28.2 ± 22.4 | 21.4 ± 20.0 | 27.3 ± 22.2 | 24.9 ± 21.5 | 0.049 |
|  | SGRQ activity | 24.0 ± 26.2 | 23.6 ± 21.8 | 37.1 ± 24.3 †¶ | 27.3 ± 24.4 | <0.001 |
|  | SGRQ impact | 13.2 ± 17.7 | 10.0 ± 13.8 | 15.3 ± 15.5 | 12.3 ± 15.5 | 0.058 |
|  | Total SGRQ | 19.2 ± 19.4 | 16.3 ± 15.2 | 24.2 ± 17.2 ¶ | 19.3 ± 17.2 | 0.007 |
|  | EQ-5D VAS score | 73.0 ± 16.3 | 74.0 ± 15.5 | 65.7 ± 19.5 ‡¶ | 71.5 ± 17.2 | 0.003 |
|  | EQ-5D utility score | 0.84 ± 0.23 | 0.91 ± 0.16 ‡ | 0.84 ± 0.18 § | 0.87 ± 0.19 | 0.006 |
| LCADL total | | 16.7 ± 6.9 | 15.2 ± 4.0 | 17.2 ± 6.9 | 16.2 ± 5.8 | 0.039 |
| Systemic biomarkers | | | | | | |
|  | CRP, log (mg/l) | 0.37 ± 0.32 | 0.52 ± 0.45 ‡ | 0.61 ± 0.42 ‡ | 0.50 ± 0.42 | 0.004 |
|  | TNF-alpha, log (pg/ml) | 0.96 ± 0.27 | 1.06 ± 0.29 | 1.07 ± 0.24 | 1.03 ± 0.27 | 0.036 |
|  | IL-6, log (pg/ml) | 0.46 ± 0.33 | 0.47 ± 0.34 | 0.53 ± 0.34 | 0.48 ± 0.34 | 0.345 |
|  | IL-8, log (pg/ml) | 0.53 ± 0.65 | 0.43 ± 0.58 | 0.44 ± 0.55 | 0.46 ± 0.59 | 0.515 |
|  | Fibrinogen, g/l | 3.31 ± 0.87 | 3.62 ± 1.13 | 3.77 ± 1.16 ‡ | 3.57 ± 1.08 | 0.029 |
|  | NOx, log (nmol/l) | 1.44 ± 0.18 | 1.44 ± 0.25 | 1.41 ± 0.23 | 1.43 ± 0.23 | 0.622 |

Values are mean ± SD or frequency. Abbreviations: BMI=body mass index; mMRC=modified Medical Research Council dyspnea scale; SGRQ=St. George’s Respiratory Questionnaire; EQ-5D=EuroQol 5 Dimensions questionnaire; VAS=Visual analogue scale; LCADL=London Chest Activities of Daily Living; CRP=C-reactive protein; TNF=tumor necrosis factor; IL=interleukin; NOx=nitrites/nitrates.

Comparisons between groups by ANOVA with Bonferroni post-hoc comparisons: † p<0.01 vs. normal weight group; ‡ p<0.05 vs. normal weight group; ¶ p<0.01 vs. overweight group; § p<0.05 vs. overweight group.

**Table S3**. Adjusted comparisons of health status, exercise tolerance and systemic inflammatory biomarkers between the study groups excluding the patients with mixed COPD-asthma phenotype

|  | | **Normal weight**  (18.5-24.9 Kg/m^2^) | **Overweight**  (25-29.9 Kg/m^2^) | **Obesity**  (>30 Kg/m^2^) | **p** |
| --- | --- | --- | --- | --- | --- |
| mMRC | | 1.60 ± 0.09 | 1.48 ± 0.07 | 1.78 ± 0.09 § | 0.041 |
| Health-related quality of life | | | | | |
|  | SGRQ symptoms | 29.1 ± 2.5 | 22.7 ± 2.0 | 24.3 ± 2.7 | 0.141 |
|  | SGRQ activity | 28.1 ± 2.5 | 24.7 ± 2.0 | 32.3 ± 2.7 § | 0.075 |
|  | SGRQ impact | 15.0 ± 1.8 | 10.7 ± 1.4 | 12.5 ± 1.9 | 0.183 |
|  | Total SGRQ | 21.6 ± 1.9 | 17.3 ± 1.5 | 20.8 ± 2.0 | 0.148 |
|  | EQ-5D VAS score | 71.8 ± 2.0 | 74.3 ± 1.6 | 67.5 ± 2.1 § | 0.037 |
|  | EQ-5D utility score | 0.84 ± 0.02 | 0.90 ± 0.02 ‡ | 0.87 ± 0.03 | 0.048 |
| LCADL total | | 16.7 ± 0.7 | 15.3 ± 0.6 | 16.4 ± 0.7 | 0.225 |
| Distance 6-min walking test, m | | 446 ± 14 | 469 ± 11 | 424 ± 15 § | 0.039 |
| Walk work, m . Kg | | 284 ± 12 | 364 ± 94 † | 389 ± 13 † | <0.001 |
| Systemic biomarkers | | | | | |
|  | CRP, log (mg/l) | 0.40 ± 0.05 | 0.53 ± 0.04 | 0.58 ± 0.06 ‡ | 0.036 |
|  | TNF-alpha, log (pg/ml) | 0.98 ± 0.04 | 1.05 ± 0.03 | 1.03 ± 0.04 | 0.385 |
|  | IL-6, log (pg/ml) | 0.49 ± 0.04 | 0.47 ± 0.04 | 0.53 ± 0.05 | 0.563 |
|  | IL-8, log (pg/ml) | 0.54 ± 0.07 | 0.42 ± 0.06 | 0.44 ± 0.08 | 0.437 |
|  | Fibrinogen, g/l | 3.44 ± 0.12 | 3.56 ± 0.10 | 3.62 ± 0.13 | 0.593 |
|  | Albumin, g/l | 45.1 ± 0.4 | 45.3 ± 0.3 | 46.2 ± 0.4 | 0.130 |
|  | NOx, log (nmol/l) | 1.45 ± 0.03 | 1.44 ± 0.02 | 1.41 ± 0.03 | 0.610 |

Values are mean ± SEM. Comparisons adjusted by gender, age, pack-year and FEV_1_ (% pred). Abbreviations: mMRC=modified Medical Research Council dyspnea scale; SGRQ=St. George’s Respiratory Questionnaire; EQ-5D=EuroQol 5 Dimensions questionnaire; VAS=Visual analogue scale; LCADL=London Chest Activities of Daily Living; CRP=C-reactive protein; TNF=tumor necrosis factor; IL=interleukin; NOx=nitrites/nitrates.

Post-hoc comparisons between groups by Bonferroni test: † p<0.001 vs. normal weight group; ‡ p<0.05 vs. normal weight group; ¶ p<0.01 vs. overweight group; § p<0.05 vs. overweight group.

**Table S4**. Adjusted risk of comorbidity and respiratory symptoms in overweight and obese COPD patients compared with normal-weight COPD patients excluding the subjects with mixed phenotype COPD-asthma. Values are adjusted for gender, age, packs-year and FEV1 (% pred.)

|  | **Overweight COPD patients** | | **Obese COPD patients** | |
| --- | --- | --- | --- | --- |
|  | **Adjusted odds ratio**  **(95% CI)** | **p** | **Adjusted odds ratio**  **(95% CI)** | **p** |
| Cardiovascular morbidity | 2.310 (0.865-6.164) | 0.095 | 4.412 (1.601-12.155) | 0.004 |
| Diabetes mellitus | 0.795 (0.263-2.410) | 0.686 | 1.857 (0.622-5.547) | 0.267 |
| Chronic cough | 0.547 (0.270-1.107) | 0.094 | 0.363 (0.151-0.875) | 0.024 |
| Chronic bronchitis | 0.281 (0.116-0.678) | 0.005 | 0.390 (0.155-0.979) | 0.045 |
| Chronic phlegm | 0.397 (0.196-0.807) | 0.011 | 0.291 (0.125-0.678) | 0.004 |
| Dyspnea | 0.960 (0.433-2.131) | 0.921 | 2.518 (1.103-5.750) | 0.028 |
| Moderate-severe dyspnea (mMRC ≥2) | 1.114 (0.552-2.249) | 0.762 | 2.862 (1.314-6.232) | 0.008 |
| Wheezing | 0.878 (0.456-1.688) | 0.695 | 1.099 (0.507-2.383) | 0.811 |
